# Good syndrome presenting with multiple pulmonary infections: a case report involving metagenomic sequencing diagnosis

**DOI:** 10.3389/fmed.2025.1649584

**Published:** 2025-08-19

**Authors:** Wenjing Zeng, Xiaoyi Feng, Binmiao Liang, Xuemei Ou

**Affiliations:** ^1^Department of Pulmonary and Critical Care Medicine, West China Hospital, Sichuan University, Chengdu, China; ^2^Department of Respiratory and Critical Care Medicine, Peking Union Medical College Hospital, Chinese Academy of Medical Sciences, Peking Union Medical College, Beijing, China

**Keywords:** good syndrome, thymoma, pulmonary infection, common variable immunodeficiency, intravenous immunoglobulin

## Abstract

Good syndrome (GS), alternatively termed thymoma with immunodeficiency, is a rare adult-onset immunodeficiency disorder characterized by concurrent thymoma and hypogammaglobulinemia, accompanied by defects in both B-cell-mediated immunity and T-cell-mediated immunity. Owing to the non-specific clinical presentation, diagnosis is frequently delayed, resulting in poor prognosis and elevated mortality. In this study, we report the case of a 69-year-old man with GS who presented with symptoms of recurrent cough and productive sputum. Metagenomic next-generation sequencing (mNGS) of oropharyngeal swabs detected multiple microorganisms, including SARS-CoV-2 (35,047 reads), Epstein–Barr virus (7,236 reads), *Micromonas pusilla* (3,674 reads), Bacillus spp. (3,284 reads), cytomegalovirus (1,203 reads), and herpes simplex virus type 1 (575 reads). Following a comprehensive clinical evaluation—including recurrent pulmonary infections, history of thymoma, and lymphopenia with immunodeficiency—the diagnosis of GS was confirmed. This patient received an intensified anti-infective regimen, with broad-spectrum carbapenem, meropenem, as the backbone therapy, combined with antifungal agents and antiviral treatment (IV ganciclovir and oral molnupiravir). After aggressive anti-infection therapy, the patient experienced clinical improvement, and chest CT demonstrated significant radiographic improvement. Although intravenous immunoglobulin (IVIG) is foundational in GS, intensive antimicrobial therapy is also critical for clinical outcomes.

## Introduction

Good syndrome (GS), first described by Robert Good in 1954, is a rare immunodeficiency disorder characterized by the co-occurrence of thymoma and immunodeficiency. Characteristic features include hypogammaglobulinemia, marked-to-absent B-cell lymphopenia, profound T-cell depletion, an inverted CD4+/CD8+ ratio, and significantly impaired mitogen-induced T-cell responses (1, 2). The onset of the condition typically occurs between the ages of 40 and 60 years. The estimated incidence of GS is 0.15 per 100,000 person-years, which is consistent with its characterization as a rare disease (3). Due to its rarity, heterogeneous clinical presentations, and lack of standardized diagnostic criteria, diagnosis is complicated, resulting in prolonged clinical courses.

We present a case of recurrent severe acute respiratory syndrome coronavirus 2 (SARS-CoV-2) infection in a patient with severe immunodeficiency due to GS.

## Case description

The clinical timeline is anchored to the index admission date (17 July 2024), with all events reported relative to this date. Approximately 6 years prior to presentation (around mid-2018), a 69-year-old man had experienced recurring episodes of cough and productive sputum, which were not medically evaluated at that time. During this period, the above symptoms recurred repeatedly. Over 4 years prior to presentation (September 2019), the patient was evaluated in the outpatient department of West China Hospital, Sichuan University. A chest CT scan revealed an anterior mediastinal mass measuring 9.0 × 4.3 × 8.0 cm, indicative of thymoma. On 15 October 2019, the patient underwent complete resection of an anterior mediastinal mass and total thymectomy via median sternotomy under general anesthesia in our hospital’s thoracic surgery department. Intraoperatively, the tumor capsule was intact with no or only mild adhesions to the surrounding tissues. The tumor and thymic tissue were completely excised. Postoperative pathology confirmed a type AB thymoma, extending to the capsule without capsular penetration. It should be emphasized that the preoperative screening for hypogammaglobulinemia was negative. A follow-up chest CT scan 2 months after surgery showed postoperative changes in the sternum, with no other abnormalities being found.

One month before admission (mid-June 2024), her respiratory symptoms worsened significantly, which manifested as progressive cough, increased sputum production, and new-onset dyspnea. Concurrent febrile episodes peaked with a temperature of 39C, prompting consultation at a tertiary hospital. Chest CT revealed bilateral scattered pulmonary infiltrates. The bronchoalveolar lavage (BAL) fluid was negative for other respiratory viruses via multiplex PCR, and microscopy for Pneumocystis was negative. Cytomegalovirus (CMV) antigen from BAL fluid was negative. SARS-CoV-2 was detected by metagenomic next-generation sequencing (mNGS) in BAL fluid (sequence number unknown).

mNGS of oropharyngeal swabs detected multiple microorganisms, including SARS-CoV-2 (35,047 reads), Epstein–Barr virus (7,236 reads), *Micromonas pusilla* (3,674 reads), *Bacillus* spp. (3,284 reads), cytomegalovirus (1,203 reads), and herpes simplex virus type 1 (575 reads). The patient was administered moxifloxacin and cefoperazone-sulbactam plus nirmatrelvir-ritonavir (SARS-CoV-2 targeted), with subcutaneous thymosin α1 (1.6 mg twice weekly) for immunomodulation and nutritional support.

This regimen resulted in significant clinical improvement, including apyrexia, resolution of cough/sputum, and diminished dyspnea. Follow-up chest CT showed considerable regression of bilateral pulmonary infiltrates. Sixteen days before admission (1 July 2024), the patient developed recurrent fever accompanied by cough and sputum production. Contrast-enhanced chest CT at Chengdu Eighth People’s Hospital (Chengdu, China) revealed bilateral multifocal consolidations suggestive of post-viral secondary infection, with concomitant postoperative changes in the sternum. Candida was cultivated by sputum culture, and the *Chlamydia pneumoniae* antibody was positive. Despite antimicrobial therapy with cefoperazone, azithromycin, voriconazole, and oseltamivir, the patient’s clinical status did not improve. Therefore, the patient was transferred to the Respiratory Department of West China Hospital of Sichuan University on the index admission date (17 July 2024) for further treatment. The patient received three doses of the BNT162b2 mRNA COVID-19 vaccine. Serological testing via chemiluminescent immunoassay (CLIA) detected no human immunodeficiency virus (HIV) antibodies prior to admission, confirming the absence of HIV infection.

**Figure 1:**
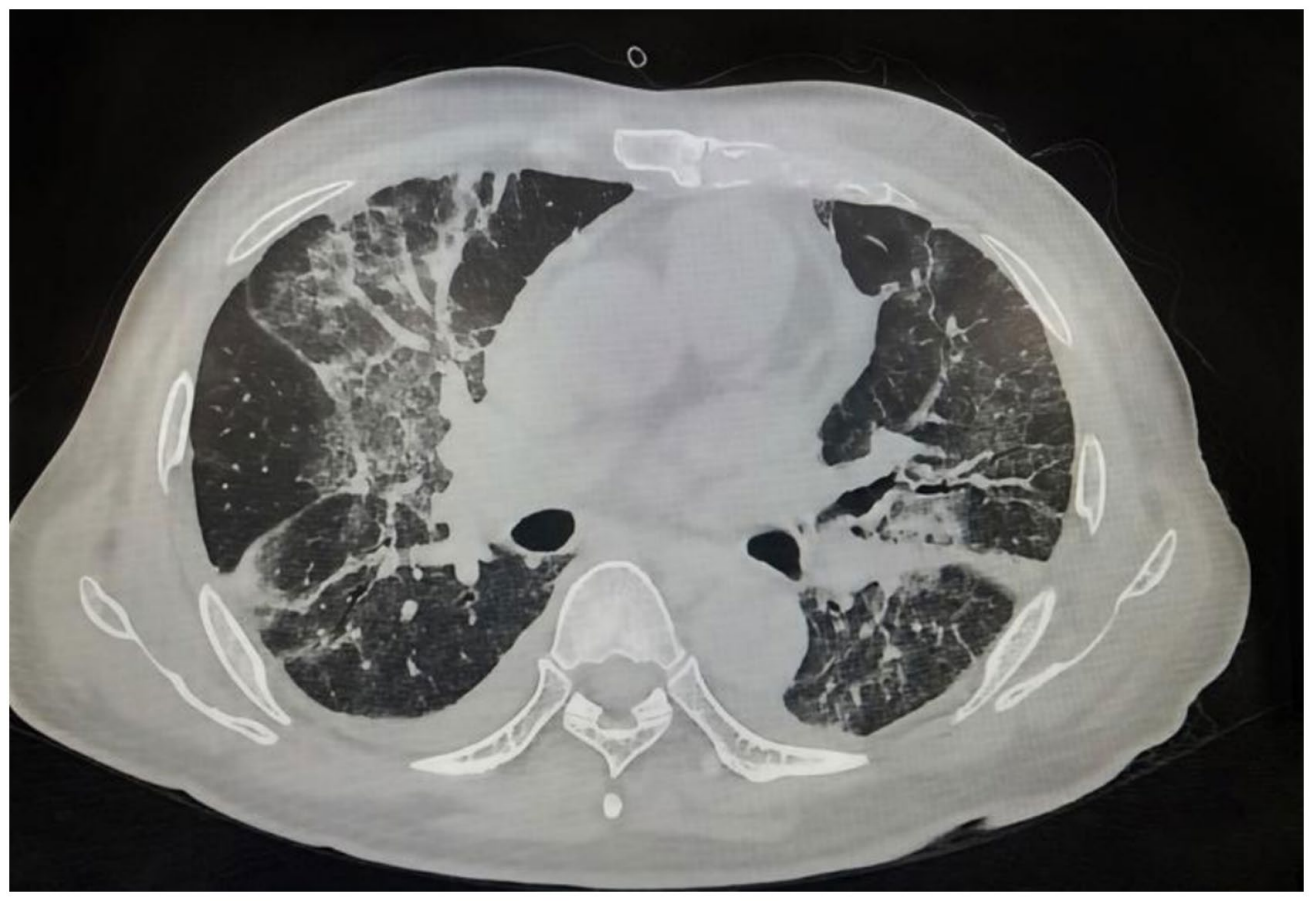
Chest CT demonstrated multifocal ground-glass opacities, linear opacities, and patchy consolidation bilaterally, suggesting an infectious etiology.

The laboratory findings are summarized in Table 1, with representative chest CT images shown in Figure 1. Comprehensive evaluation of medical history, clinical manifestations, and ancillary investigations established the diagnosis of GS, manifested by (1) recurrent pulmonary infections; (2) histologically confirmed thymoma; and (3) lymphopenia featuring B-cell aplasia (CD19^⁺^0 cells/μL), with hypogammaglobulinemia (IgG 3.03 g/L), profound CD4^⁺^T-lymphocytopenia (83 cells/μL), and CD3^⁺^T-cell depletion (167 cells/μL). Combination antimicrobial therapy was initiated immediately upon hospitalization: meropenem (1 g intravenous infusion every 8 h for 14 days), ganciclovir (0.25 g intravenous infusion every 24 h for 14 days), voriconazole (0.2 g taken orally twice daily for 14 days), molnupiravir (0.8 g taken orally twice daily for 5 days), and voriconazole (0.2 g taken orally twice daily for 14 days).

**Table 1:** Selected hematological and immunological indices of the patients.

Date	2024/7/17	2024/7/20	2024/7/25	Reference interval
HGB (g/L)	82	86	105	130–175
RBC (10*12/L)	3.49	3.76	4.46	4.3–5.8
WBC (10*12/L)	4.39	6.69	3.15	3.5–9.5
PLT (10*9/L)	270	295	264	100–300
NE (10*9/L)	3.93	5.97	2.19	1.8–6.3
LYM (10*9/L)	0.2	0.29	0.62	1.1–3.2
B-cell Count (cell/uL)	0	—	—	63–100
T cell (CD3) (%)	85.89	—	—	48.60–81.01
CD3 count (cell/uL)	167	—	—	560–2,476
CD4 count (cell/uL)	83	—	—	338–1,125
CD8 count (cell/uL)	84	—	—	184–1,126
CD4/CD8	0.98	—	—	0.71–3.40
NK count (cell/uL)	21	—	—	133–1,228
IGG (g/L)	3.03	—	—	8.6–17.4
IGA (g/L)	0.094	—	—	1.0–4.2
IGM (g/L)	0.109	—	—	0.3–2.2
ALT (U/L)	113	162	59	<50
AST (U/L)	98	99	28	<40
ALB (g/L)	29.7	30.6	36.5	40–55
GLB (g/L)	15.2	17.1	17.1	20–40
CRP (mg/L)	173	70.6	9.25	0–6.0
PCT (ng/L)	0.119	0.055	0.021	<0.046
EBV-DNA PCR (Copies/mL)	Negative (No Amplification)			Negative (No Amplification)
HCMV-DNA PCR (Copies/mL)	1.99E+02			Negative (No Amplification)

HGB, hemoglobin; RBC, erythrocyte count; WBC, white blood cell count; PLT, platelet count; NE, neutrophil count; LYM, lymphocyte count; B cell, B lymphocyte; T cell, T lymphocyte; CD3, cluster of differentiation 3; CD4, cluster of differentiation 4; CD8, cluster of differentiation 8; NK, natural killer cell; IGG, immunoglobulin G; IGA, immunoglobulin A; IGM, immunoglobulin M; ALT, alanine aminotransferase; AST, aspartate aminotransferase; ALB, albumin; GLB, globulin; CRP, C-reactive protein; PCT, procalcitonin; EBV-DNA qPCR, Epstein–Barr virus DNA quantitative real-time polymerase chain reaction; HCMV-DNA qPCR, human cytomegalovirus DNA quantitative real-time polymerase chain reaction.

**Figure 2:**
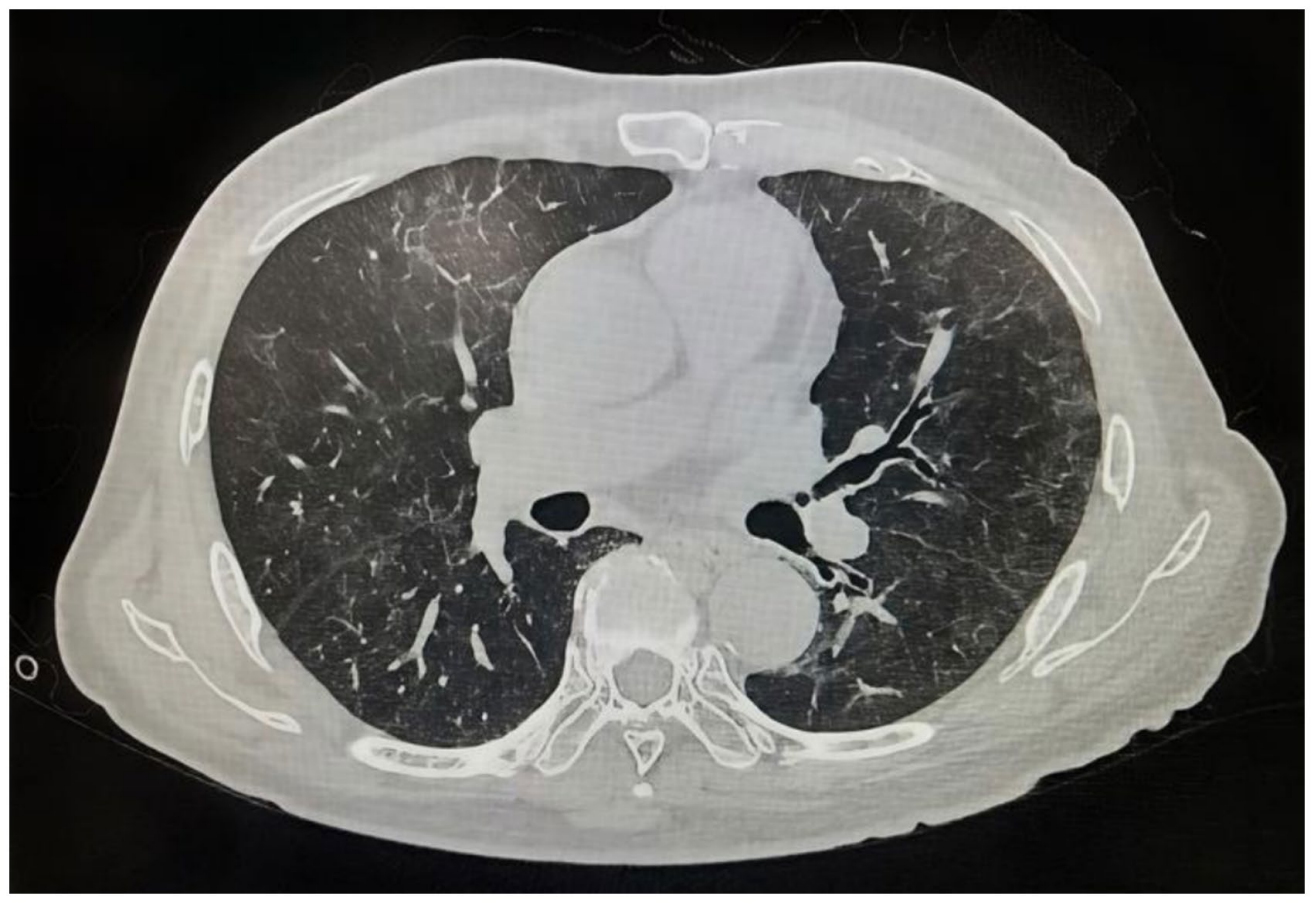
Marked resolution of bilateral pulmonary infectious lesions is seen compared to prior imaging.

Adjunctive therapies included anticoagulation, hepatoprotective agents, diuretics, gastroprotective agents, antispasmodics, muco-kinetics, nebulized therapy, and supplemental oxygen administration. During hospitalization, intravenous immunoglobulin therapy was discussed with the family; however, due to personal considerations of the patient and family, they requested to postpone this treatment until after transfer to a local hospital. Following the above treatment, the patient’s temperature returned to normal, and their cough and dyspnea symptoms improved. Pulmonary auscultation revealed the disappearance of moist crackles. Repeat SARS-CoV-2 nucleic acid testing returned negative, and chest CT demonstrated significant radiographic improvement in pulmonary infiltrates compared to prior imaging (Figure 2). Post-discharge management consisted of oral erdosteine for sputum clearance. Intravenous immunoglobulin (IVIG) replacement therapy was recommended for consideration during subsequent follow-up evaluations.

Three months after discharge, surveillance chest CT demonstrated complete resolution of the pulmonary lesions. The patient initiated regular IVIG maintenance therapy locally but was subsequently lost to follow-up because of patient-related factors.

## Discussion

We treated a patient with recurrent SARS-CoV-2 infection, wherein multiple virus mutations had accumulated due to severe immunodeficiency resulting from GS. The immunopathology of GS is defined by thymic microenvironment disruption, B-cell deficiency (resulting in hypogammaglobulinemia and the absence of antibody-secreting plasma cells), and dysregulated T-cell homeostasis. In patients with GS who are reinfected with SARS-CoV-2, preexisting B lymphocyte differentiation defects and hypogammaglobulinemia substantially impair vaccine-induced humoral immunity. This results in deficient neutralizing antibody generation and compromised memory B-cell reservoirs. Concurrent CD4+ T-lymphocytopenia and restricted T-cell receptor diversity further attenuate cellular immune responses. The host fails to mount effective recall immunity during reinfection, manifested by suboptimal antigen-specific antibody boosting, delayed T-cell activation kinetics, and dysregulated cytokine profiles. Such primary immunodeficiency severely impedes viral clearance, driving persistently high viral loads, protracted symptomatology, and significantly heightened secondary infection risks (4–6). The patient described in this study presented with co-infection of SARS-CoV-2, EBV, CMV, HSV-1, and Candida species. Comprehensive immunological analysis demonstrated that the heightened susceptibility to CMV, EBV, and candidiasis in GS stems from three cardinal immunodeficiencies: (1) cellular immunosurveillance failure: CD8⁺T-cell exhaustion and NK-cell dysfunction impair viral control, reactivating latent CMV/EBV and facilitating herpesvirus spread; (2) humoral defense collapse: B-cell deficiency ablates neutralizing antibodies, enabling pathogen dissemination; and (3) mucosal barrier breakdown: Th17 pathway suppression reduces IL-17, while secretory IgA deficiency permits Candida epithelial invasion and viral–fungal coinfections (4, 7, 8). This finding significantly increases the risk of death from opportunistic infections.

GS manifests as recurrent infections—predominantly pneumonia (cough, sputum, dyspnea, and fever), severe diarrhea, and sinusitis—affecting single or multiple organ systems. Research on 160 patients with GS found that infections were recorded in 150 patients (92.6%), and the leading site was the sinopulmonary tract (67.3%). Other common sites of infection included the gastrointestinal tract system (27.3%), skin and soft tissue (18.0%), mucosa (14.0%), and eye (10.0%). The most frequently recorded bacteria, fungi, and viruses were *Pseudomonas* spp. (12.7%), *Candida* spp. (16.7%), and cytomegalovirus (24.7%), respectively (9, 10). In addition to recurrent infections, patients with GS are often complicated by autoimmune diseases, with simple aplastic anemia (31.3%), myasthenia gravis (27.7%), and lichen planus (22.9%) being among the more common manifestations (11, 12). In this study, the patient presented with polymicrobial pulmonary infection as the primary presentation, involving fungal, viral, and bacterial pathogens. Notably, autoimmune comorbidities—predominantly myasthenia gravis, pure red cell aplasia, pernicious anemia, and immune thrombocytopenia—are prevalent in GS, collectively contributing to its substantial disease burden and mortality risk.

Since GS is uncommon and manifests primarily as sporadic occurrences, there are no established clinical diagnostic standards. The current accepted diagnostic criteria are as follows: (1) recurrent infections involving the respiratory tract, gastrointestinal tract, or urinary tract. Additionally, patients may develop comorbid autoimmune disorders, such as myasthenia gravis; (2) a history of thymoma; (3) laboratory examination indices: hypogammaglobulinemia (decreased IgG, IgA, and IgM), decreased or completely lacking B lymphocytes in peripheral blood, reduced NK cell counts, and T lymphocyte dysfunction; and (4) the exclusion of phenocopies such as common variable immunodeficiency (CVID) and X-linked agammaglobulinemia (XLA) is essential, given their similar manifestations of recurrent infections and humoral immune deficiency (2, 13, 14). The patient presented with recurrent pulmonary infections and a histologically confirmed thymoma. Laboratory investigations revealed the following: (1) complete B-cell absence (0 cells/μL) with hypogammaglobulinemia (IgG 3.03 g/L), indicating humoral immunodeficiency; (2) depletion of CD4⁺ T-lymphocytes (83 cells/μL) and total CD3⁺ T-cell (167 cells/μL), indicating compromised cellular immunity; and (3) severe NK-cell deficiency (21 cells/μL), which may increase the risk of EBV-driven malignancies. Taken together, the diagnosis of GS was definitively established. The non-specific clinical manifestations and extremely low incidence of GS pose significant diagnostic challenges, often resulting in a protracted clinical course and diagnostic delays for patients. In this case, the patient experienced a 6-year diagnostic delay from symptom onset to definitive diagnosis, characterized by recurrent severe infections. Consequently, in clinical practice, patients with a history of thymoma and recurrent infections should prompt the consideration of GS. Prompt initiation of immunological screening, including quantitative serum immunoglobulin assays and lymphocyte subset analysis, is essential to facilitate early diagnosis and intervention, thereby reducing the risk of multiorgan damage and mortality.

The treatments for GS are as follows: (1) intravenous immunoglobulin (IVIG) serves as a key therapeutic intervention to reduce infection risk and improve immune function; (2) thymoma excision did not reverse immune dysfunction. However, previous studies have shown that, although it may not reverse the immune abnormality of GS, clinical symptoms improve in some patients; and (3) anti-infection treatment, which is very important for determining the kind of pathogen infection and targeted anti-infective treatment (15–17). Although IVIG serves as the cornerstone therapy for GS, intensive anti-infective treatment is also critical during the acute phase of infection. The patient was admitted with a mixed pulmonary infection. Based on his history of recurrent infections, further immunological evaluation—including serum immunoglobulin quantification and lymphocyte subset analysis—was performed, ultimately confirming the diagnosis of GS. This case received an intensified anti-infective regimen: broad-spectrum carbapenem, i.e., meropenem, as the backbone therapy, combined with antifungal agents and antiviral treatment (intravenous infusion ganciclovir and oral molnupiravir). Molnupiravir is an oral antiviral targeting SARS-CoV-2 infection, while ganciclovir—a viral DNA polymerase inhibitor—serves as first-line therapy for CMV pneumonitis and is prioritized over foscarnet (higher nephrotoxicity) in non-severe cases. Despite the availability of novel anti-CMV agents such as maribavir, ganciclovir was retained in this study based on cost accessibility in resource-limited settings and well-documented safety evidence accumulated in renal impairment patients (18–21). Notably, significant clinical and radiographic improvement occurred without concomitant IVIG. This finding indicates that prompt initiation of broad-spectrum anti-infective therapy (covering Gram-negative bacteria, fungi, and viruses) in GS patients is essential for improving survival outcomes.

Patients with GS frequently develop progressively severe opportunistic infections due to T-cell dysfunction, combined with B-lymphocyte deficiency, and is associated with an adverse prognosis. One study revealed that the overall mortality rate of GS is 46%, and the 5-year survival rate is approximately 70%, and the 10-year survival rate is 33% (22). Moreover, Jansen followed 39 patients and reported that 5-year and 10-year survival rates were 82 and 68%, respectively (12, 23). The primary causes of mortality in this population are life-threatening infections and multiorgan dysfunction syndrome (MODS) secondary to immune deficiency.

While this case provides valuable insights into the diagnostic challenges and initial management approaches for GS, a key limitation remains the absence of follow-up data after IVIG therapy. This gap precludes the assessment of clinical trajectory, treatment response, and potential adverse events, thereby constraining conclusions on IVIG’s long-term therapeutic role in this rare disorder.

## Conclusion

In conclusion, GS represents an adult-onset immunodeficiency defined by thymoma and B-cell depletion. In clinical practice, patients with a history of thymoma and recurrent opportunistic infections should be highly suspected of having GS. Therapeutically, in addition to thymectomy and IVIG replacement, anti-infective therapy is critical to improving clinical outcomes.

## Data Availability

The datasets presented in this study can be found in online repositories. The names of the repository/repositories and accession number(s) can be found in the article/supplementary material.
